# 6-Meth­oxy-4-(2,4,5-tri­meth­oxy­phen­yl)-2,2′-bi­pyridine-5-carbo­nitrile

**DOI:** 10.1107/S1600536813023891

**Published:** 2013-09-04

**Authors:** Suchada Chantrapromma, Thitipone Suwunwong, Pumsak Ruanwas, Ching Kheng Quah, Hoong-Kun Fun

**Affiliations:** a Department of Chemistry, Faculty of Science, Prince of Songkla University, Hat-Yai, Songkhla 90112, Thailand; b X-ray Crystallography Unit, School of Physics, Universiti Sains Malaysia, 11800 USM, Penang, Malaysia

## Abstract

In the title 3-cyano­pyridine derivative, C_21_H_19_N_3_O_4_, the 3-cyano-substituted pyridine ring forms dihedral angles of 2.35 (5) and 41.60 (5)° with the unsubstituted pyridine and 2,4,5-trimeth­oxy-substituted benzene rings, respectively. The dihedral angle between the unsubstituted pyridine and benzene rings is 39.84 (5)°. The meth­oxy groups form C_meth­yl_—O—C—(C,N) torsion angles in the range 0.80 (15)–11.45 (15)°. In the crystal, mol­ecules related by 2_1_ screw axes are linked by weak C—H⋯N hydrogen bonds along [010]. In addition, weak C—H⋯π inter­actions and π–π stacking inter­actions between pyridine rings, with a centroid–centroid distance of 3.6448 (6) Å, are observed.

## Related literature

For the synthesis and applications of 3-cyano­pyridine derivatives, see: Al-Jaber *et al.* (2012 ▶); Brandt *et al.* (2010 ▶); El-Sayed *et al.* (2011 ▶); Ji *et al.* (2007 ▶); Kim *et al.* (2005 ▶); Koner *et al.* (2012 ▶); Suwunwong *et al.* (2011 ▶); Zhou *et al.* (2006 ▶). For related structures, see: Chantrapromma *et al.* (2010 ▶); Suwunwong *et al.* (2012 ▶). For standard bond-length data, see: Allen *et al.* (1987 ▶).
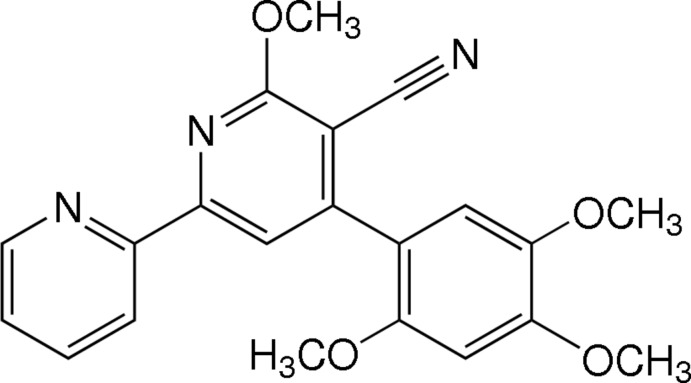


## Experimental

### 

#### Crystal data

C_21_H_19_N_3_O_4_
*M* *_r_* = 377.39
Monoclinic, 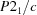

*a* = 14.9967 (3) Å
*b* = 7.4039 (2) Å
*c* = 17.5795 (4) Å
β = 114.080 (1)°
*V* = 1782.06 (7) Å^3^
*Z* = 4
Mo *K*α radiation
μ = 0.10 mm^−1^
*T* = 100 K
0.60 × 0.29 × 0.23 mm

#### Data collection

Bruker APEXII CCD area-detector diffractometer
Absorption correction: multi-scan (*SADABS*; Bruker, 2005 ▶) *T*_min_ = 0.943, *T*_max_ = 0.977
20323 measured reflections
5182 independent reflections
4386 reflections with *I* > 2σ(*I*)
*R*_int_ = 0.030

#### Refinement

*R*[*F* ^2^ > 2σ(*F* ^2^)] = 0.042
*wR*(*F* ^2^) = 0.116
*S* = 1.03
5182 reflections
257 parameters
H-atom parameters constrained
Δρ_max_ = 0.40 e Å^−3^
Δρ_min_ = −0.31 e Å^−3^

### 

Data collection: *APEX2* (Bruker, 2005 ▶); cell refinement: *SAINT* (Bruker, 2005 ▶); data reduction: *SAINT*; program(s) used to solve structure: *SHELXTL* (Sheldrick, 2008 ▶); program(s) used to refine structure: *SHELXTL*; molecular graphics: *SHELXTL*; software used to prepare material for publication: *SHELXTL*, *PLATON* (Spek, 2009 ▶), *Mercury* (Macrae *et al.*, 2006 ▶) and *publCIF* (Westrip, 2010 ▶).

## Supplementary Material

Crystal structure: contains datablock(s) global, I. DOI: 10.1107/S1600536813023891/lh5641sup1.cif

Structure factors: contains datablock(s) I. DOI: 10.1107/S1600536813023891/lh5641Isup2.hkl

Supplementary material file. DOI: 10.1107/S1600536813023891/lh5641Isup3.cml

Additional supplementary materials:  crystallographic information; 3D view; checkCIF report

## Figures and Tables

**Table 1:** Hydrogen-bond geometry (Å, °) *Cg*_3_ is the centroid of the C11–C16 ring.

*D*—H⋯*A*	*D*—H	H⋯*A*	*D*⋯*A*	*D*—H⋯*A*
C20—H20*C*⋯N3^i^	0.98	2.59	3.3774 (17)	138
C1—H1*A*⋯*Cg*3^ii^	0.95	2.89	3.7062 (13)	145

Symmetry codes: (i) 
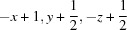
; (ii) 
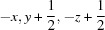
.

**Fig. 1.**
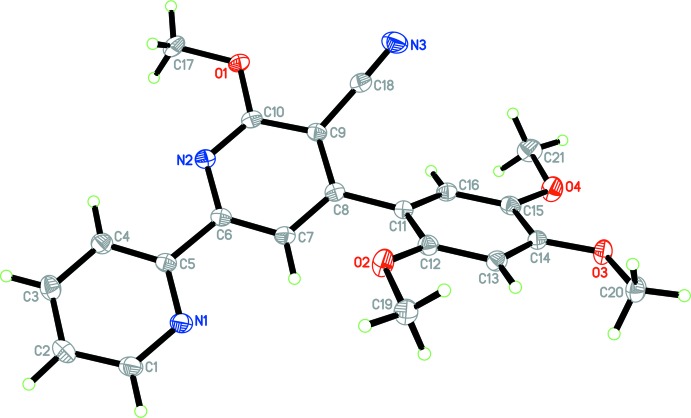
The molecular structure of the title compound, showing 50% probability displacement ellipsoids.

**Fig. 2.**
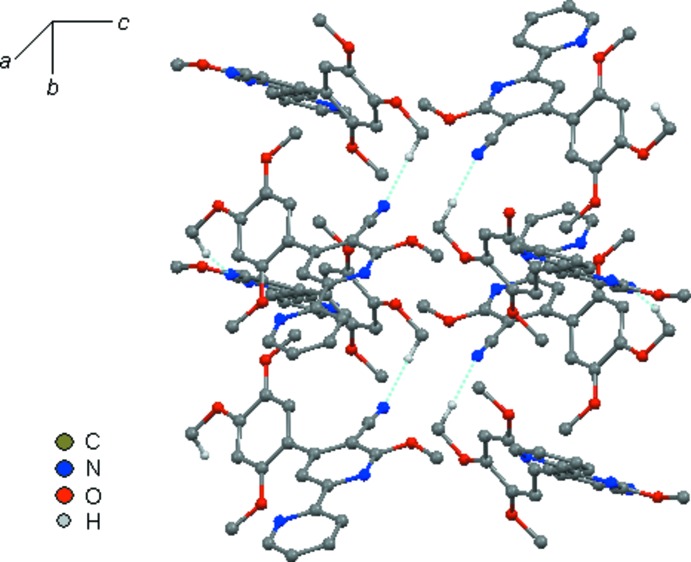
The crystal packing of the title compound viewed approximately along the a axis. Weak C—H···N interactions are shown as dashed lines. For clarity, only H atoms involved in hydrogen bonds are shown.

## supplementary crystallographic information

### 1. Comment

3-Cyanopyridine derivatives have been reported for their wide range of applications such as in antimicrobial, analgesic, anti-hyperglycemic, antiproliferative and antitumor activities (Brandt *et al.*, 2010; El-Sayed *et al.*, 2011; Ji *et al.*, 2007; Kim *et al.*, 2005), as well as in usage as fluorescence materials (Koner *et al.*, 2012). There are several methods to synthesize substituted 3-cyanopyridine derivatives including by condensation of α,β-unsaturated ketones and malononitrile (Al-Jaber *et al.*, 2012; Zhou *et al.*, 2006). Our research is aimed at the synthesis and preliminary fluorescent and antibacterial screening of the 3-cyanopyridine derivatives. The title compound (I) was synthesized and was found to exhibit fluorescence property which will be reported elsewhere together with the other related compounds. Herein the crystal structure of (I) was reported.

The title compound (I), C_21_H_19_N_3_O_4_ is a non-planar molecule (Fig. 1) in which the 3-cyano-substituted pyridine ring is approximately co-planar with the unsubstituted pyridine ring and inclined to the 2,4,5-trimethoxy-substituted ring with dihedral angles of 2.35 (5) and 41.60 (5)°, respectively. The dihedral angle between the unsubstituted pyridine and benzene rings is 39.84 (5)°. For the 2,4,5-trimethoxyphenyl moiety, the two substituted methoxy groups at *ortho* and *meta* positions are slightly twisted with the attached benzene ring with torsion angles C19–O2–C12–C13 = -11.45 (15)° and C21–O4–C15–C16 = -8.11 (16)° whereas the *para* methoxy group is essentially co-planar with a dihedral angle of C20–O3–C14–C13 = -0.80 (15)°. In addition, the methoxy group of the 3-cyanopyridine group is also essentially co-planar with the pyridine ring as indicated by the torsion angle C17–O1–C10–N2 = 6.18 (14)°. The bond distances agree with the literature values (Allen *et al.*, 1987) and are comparable with those found in the related structures (Chantrapromma *et al.*, 2010; Suwunwong *et al.*, 2012).

In the crystal (Fig. 2), molecules related by 2_1_ screw axes are linked by weak C—H···N interactions (Table 1) to form helical chains. The crystal is further stabilized by weak C—H···π interactions (Table 1) and π–π interaction with the *Cg*_1_···*Cg*_2_^iii^ distance of 3.6448 (6) Å (iii = -*x*, 1 - *y*, -*z*); *Cg*_1_ and *Cg*_2_ are the centroids of N1/C1–C5 and N2/C6–C10 pyridine rings, respectively.

### 2. Experimental

The title compound (I) was synthesized by stirring a solution of (*E*)-1-(pyridin-2-yl)-3-(2,4,5-trimethoxyphenyl)prop-2-en-1-one (Suwunwong *et al.*, 2011) (0.30 g, 1 mmol) in 10 ml of methanol with a freshly prepared sodium methoxide (1 mmol of sodium in 20 ml of methanol). Excess malononitrile (0.13 g, 2 mmol) was then added with continuous stirring at room temperature until the precipitate was separated out. The resulting solid was filtered and washed with hexane. Colorless block-shaped single crystals of the title compound suitable for *x*-ray structure determination were recrystallized from ethanol/methanol (1:1 *v*/*v*) by the slow evaporation of the solvent at room temperature after several days, Mp. 506–507 K.

### 3. Refinement

All H atoms were positioned geometrically and allowed to ride on their parent atoms, with d(C—H) = 0.95 Å for aromatic and 0.98 Å for CH_3_ atoms. The *U*_iso_ values were constrained to be 1.5*U*_eq_ of the carrier atom for methyl H atoms and 1.2*U*_eq_ for the remaining H atoms. A rotating group model was used for the methyl groups.

### Crystal data

**Table:**

C_21_H_19_N_3_O_4_	*F*(000) = 792
*M**_r_* = 377.39	*D*_x_ = 1.407 Mg m^−^^3^
Monoclinic, *P*2_1_/*c*	Melting point = 506–507 K
Hall symbol: -P 2ybc	Mo *K*α radiation, λ = 0.71073 Å
*a* = 14.9967 (3) Å	Cell parameters from 5182 reflections
*b* = 7.4039 (2) Å	θ = 2.4–30.0°
*c* = 17.5795 (4) Å	µ = 0.10 mm^−^^1^
β = 114.080 (1)°	*T* = 100 K
*V* = 1782.06 (7) Å^3^	Block, colorless
*Z* = 4	0.60 × 0.29 × 0.23 mm

### Data collection

**Table:**

Bruker APEXII CCD area-detector diffractometer	5182 independent reflections
Radiation source: sealed tube	4386 reflections with *I* > 2σ(*I*)
Graphite monochromator	*R*_int_ = 0.030
φ and ω scans	θ_max_ = 30.0°, θ_min_ = 2.4°
Absorption correction: multi-scan (*SADABS*; Bruker, 2005)	*h* = −21→21
*T*_min_ = 0.943, *T*_max_ = 0.977	*k* = −10→10
20323 measured reflections	*l* = −22→24

### Refinement

**Table:**

Refinement on *F*^2^	Primary atom site location: structure-invariant direct methods
Least-squares matrix: full	Secondary atom site location: difference Fourier map
*R*[*F*^2^ > 2σ(*F*^2^)] = 0.042	Hydrogen site location: inferred from neighbouring sites
*wR*(*F*^2^) = 0.116	H-atom parameters constrained
*S* = 1.03	*w* = 1/[σ^2^(*F*_o_^2^) + (0.0629*P*)^2^ + 0.5246*P*] where *P* = (*F*_o_^2^ + 2*F*_c_^2^)/3
5182 reflections	(Δ/σ)_max_ = 0.001
257 parameters	Δρ_max_ = 0.40 e Å^−^^3^
0 restraints	Δρ_min_ = −0.31 e Å^−^^3^

### Special details

**Table:**

Experimental. The crystal was placed in the cold stream of an Oxford Cryosystems Cobra open-flow nitrogen cryostat (Cosier & Glazer, 1986) operating at 100.0 (1) K.
Geometry. All e.s.d.'s (except the e.s.d. in the dihedral angle between two l.s. planes) are estimated using the full covariance matrix. The cell e.s.d.'s are taken into account individually in the estimation of e.s.d.'s in distances, angles and torsion angles; correlations between e.s.d.'s in cell parameters are only used when they are defined by crystal symmetry. An approximate (isotropic) treatment of cell e.s.d.'s is used for estimating e.s.d.'s involving l.s. planes.
Refinement. Refinement of *F*^2^ against ALL reflections. The weighted *R*-factor *wR* and goodness of fit *S* are based on *F*^2^, conventional *R*-factors *R* are based on *F*, with *F* set to zero for negative *F*^2^. The threshold expression of *F*^2^ > σ(*F*^2^) is used only for calculating *R*-factors(gt) *etc*. and is not relevant to the choice of reflections for refinement. *R*-factors based on *F*^2^ are statistically about twice as large as those based on *F*, and *R*- factors based on ALL data will be even larger.

### Fractional atomic coordinates and isotropic or equivalent isotropic displacement parameters (Å^2^)

**Table:**

	*x*	*y*	*z*	*U*_iso_*/*U*_eq_	
O1	0.08878 (6)	0.11337 (11)	−0.09750 (5)	0.01663 (17)	
O2	0.21018 (6)	0.46941 (10)	0.24541 (5)	0.01852 (17)	
O3	0.48961 (5)	0.12019 (10)	0.42643 (5)	0.01676 (17)	
O4	0.44854 (6)	−0.13384 (11)	0.31664 (5)	0.02014 (18)	
N1	−0.10990 (7)	0.40828 (13)	0.10405 (6)	0.01619 (19)	
N2	−0.00502 (6)	0.22953 (12)	−0.03257 (5)	0.01373 (18)	
N3	0.32255 (7)	0.04540 (15)	0.03661 (7)	0.0231 (2)	
C1	−0.19408 (8)	0.48087 (15)	0.09883 (7)	0.0174 (2)	
H1A	−0.1982	0.5191	0.1489	0.021*	
C2	−0.27560 (8)	0.50345 (15)	0.02446 (7)	0.0176 (2)	
H2A	−0.3340	0.5543	0.0239	0.021*	
C3	−0.26961 (8)	0.44990 (16)	−0.04895 (7)	0.0187 (2)	
H3A	−0.3240	0.4640	−0.1009	0.022*	
C4	−0.18290 (8)	0.37531 (15)	−0.04529 (7)	0.0160 (2)	
H4A	−0.1768	0.3381	−0.0947	0.019*	
C5	−0.10496 (7)	0.35617 (13)	0.03249 (6)	0.01291 (19)	
C6	−0.01002 (7)	0.27984 (13)	0.03969 (6)	0.01266 (19)	
C7	0.06802 (7)	0.26430 (14)	0.11670 (6)	0.0139 (2)	
H7A	0.0605	0.2991	0.1658	0.017*	
C8	0.15792 (7)	0.19739 (13)	0.12237 (6)	0.01287 (19)	
C9	0.16324 (7)	0.14605 (14)	0.04744 (6)	0.01298 (19)	
C10	0.07884 (7)	0.16495 (14)	−0.02772 (6)	0.01309 (19)	
C11	0.24309 (7)	0.18194 (14)	0.20364 (6)	0.01303 (19)	
C12	0.26734 (7)	0.31794 (14)	0.26440 (6)	0.0139 (2)	
C13	0.34940 (7)	0.30003 (14)	0.33993 (6)	0.0140 (2)	
H13A	0.3648	0.3925	0.3808	0.017*	
C14	0.40836 (7)	0.14785 (14)	0.35544 (6)	0.01330 (19)	
C15	0.38513 (7)	0.00954 (14)	0.29540 (7)	0.0144 (2)	
C16	0.30335 (7)	0.02751 (14)	0.22149 (6)	0.01395 (19)	
H16A	0.2872	−0.0669	0.1815	0.017*	
C17	0.00747 (8)	0.15056 (17)	−0.17547 (7)	0.0197 (2)	
H17A	0.0223	0.1065	−0.2216	0.030*	
H17B	−0.0042	0.2811	−0.1813	0.030*	
H17C	−0.0510	0.0894	−0.1766	0.030*	
C18	0.25207 (8)	0.08862 (14)	0.04235 (7)	0.0156 (2)	
C19	0.22509 (8)	0.59686 (15)	0.31086 (7)	0.0179 (2)	
H19A	0.1754	0.6919	0.2905	0.027*	
H19B	0.2901	0.6508	0.3286	0.027*	
H19C	0.2199	0.5350	0.3582	0.027*	
C20	0.51581 (8)	0.25983 (15)	0.48834 (7)	0.0176 (2)	
H20A	0.5748	0.2240	0.5366	0.026*	
H20B	0.4622	0.2786	0.5057	0.026*	
H20C	0.5284	0.3722	0.4649	0.026*	
C21	0.43561 (9)	−0.26391 (16)	0.25325 (8)	0.0222 (2)	
H21A	0.4884	−0.3531	0.2740	0.033*	
H21B	0.4369	−0.2032	0.2042	0.033*	
H21C	0.3727	−0.3249	0.2380	0.033*	

### Atomic displacement parameters (Å^2^)

**Table:**

	*U*^11^	*U*^22^	*U*^33^	*U*^12^	*U*^13^	*U*^23^
O1	0.0182 (4)	0.0217 (4)	0.0112 (3)	0.0019 (3)	0.0073 (3)	−0.0016 (3)
O2	0.0206 (4)	0.0147 (4)	0.0154 (4)	0.0058 (3)	0.0025 (3)	−0.0031 (3)
O3	0.0152 (3)	0.0164 (4)	0.0146 (4)	0.0022 (3)	0.0019 (3)	−0.0016 (3)
O4	0.0214 (4)	0.0175 (4)	0.0186 (4)	0.0082 (3)	0.0052 (3)	−0.0020 (3)
N1	0.0170 (4)	0.0174 (4)	0.0164 (4)	0.0018 (3)	0.0091 (3)	0.0008 (3)
N2	0.0147 (4)	0.0135 (4)	0.0135 (4)	−0.0011 (3)	0.0063 (3)	−0.0006 (3)
N3	0.0234 (5)	0.0244 (5)	0.0266 (5)	0.0047 (4)	0.0154 (4)	0.0035 (4)
C1	0.0202 (5)	0.0171 (5)	0.0187 (5)	0.0027 (4)	0.0119 (4)	0.0012 (4)
C2	0.0152 (5)	0.0150 (5)	0.0248 (6)	0.0012 (4)	0.0104 (4)	0.0006 (4)
C3	0.0143 (4)	0.0197 (5)	0.0196 (5)	0.0000 (4)	0.0043 (4)	−0.0017 (4)
C4	0.0160 (4)	0.0168 (5)	0.0152 (5)	−0.0007 (4)	0.0062 (4)	−0.0025 (4)
C5	0.0141 (4)	0.0111 (4)	0.0153 (5)	−0.0013 (3)	0.0077 (4)	0.0000 (3)
C6	0.0142 (4)	0.0110 (4)	0.0144 (5)	−0.0008 (3)	0.0075 (4)	−0.0004 (3)
C7	0.0150 (4)	0.0148 (4)	0.0135 (5)	−0.0001 (4)	0.0074 (4)	−0.0011 (3)
C8	0.0141 (4)	0.0120 (4)	0.0129 (4)	−0.0006 (3)	0.0059 (4)	−0.0001 (3)
C9	0.0139 (4)	0.0122 (4)	0.0142 (5)	0.0003 (3)	0.0072 (4)	−0.0002 (3)
C10	0.0167 (4)	0.0119 (4)	0.0120 (4)	−0.0006 (3)	0.0073 (4)	−0.0005 (3)
C11	0.0128 (4)	0.0144 (5)	0.0125 (4)	0.0001 (3)	0.0058 (3)	0.0000 (3)
C12	0.0140 (4)	0.0135 (5)	0.0146 (5)	0.0013 (4)	0.0061 (4)	0.0005 (4)
C13	0.0152 (4)	0.0136 (5)	0.0134 (5)	0.0001 (4)	0.0059 (4)	−0.0011 (4)
C14	0.0123 (4)	0.0152 (5)	0.0124 (4)	−0.0005 (3)	0.0051 (4)	0.0014 (3)
C15	0.0151 (4)	0.0130 (5)	0.0167 (5)	0.0022 (4)	0.0083 (4)	0.0010 (4)
C16	0.0158 (4)	0.0136 (5)	0.0135 (5)	−0.0004 (4)	0.0071 (4)	−0.0016 (4)
C17	0.0215 (5)	0.0256 (6)	0.0107 (5)	−0.0002 (4)	0.0051 (4)	0.0000 (4)
C18	0.0183 (5)	0.0152 (5)	0.0143 (5)	0.0003 (4)	0.0078 (4)	0.0011 (4)
C19	0.0188 (5)	0.0158 (5)	0.0172 (5)	0.0028 (4)	0.0055 (4)	−0.0041 (4)
C20	0.0156 (5)	0.0177 (5)	0.0162 (5)	−0.0018 (4)	0.0030 (4)	−0.0029 (4)
C21	0.0273 (6)	0.0188 (5)	0.0225 (6)	0.0059 (4)	0.0122 (5)	−0.0033 (4)

### Geometric parameters (Å, º)

**Table:**

O1—C10	1.3499 (12)	C8—C9	1.4041 (14)
O1—C17	1.4415 (13)	C8—C11	1.4820 (13)
O2—C12	1.3676 (12)	C9—C10	1.4152 (13)
O2—C19	1.4332 (13)	C9—C18	1.4355 (14)
O3—C14	1.3576 (12)	C11—C12	1.4036 (14)
O3—C20	1.4349 (13)	C11—C16	1.4110 (14)
O4—C15	1.3715 (12)	C12—C13	1.4006 (14)
O4—C21	1.4250 (14)	C13—C14	1.3888 (14)
N1—C1	1.3403 (14)	C13—H13A	0.9500
N1—C5	1.3465 (13)	C14—C15	1.4085 (14)
N2—C10	1.3152 (13)	C15—C16	1.3819 (14)
N2—C6	1.3549 (13)	C16—H16A	0.9500
N3—C18	1.1476 (14)	C17—H17A	0.9800
C1—C2	1.3883 (15)	C17—H17B	0.9800
C1—H1A	0.9500	C17—H17C	0.9800
C2—C3	1.3876 (16)	C19—H19A	0.9800
C2—H2A	0.9500	C19—H19B	0.9800
C3—C4	1.3899 (15)	C19—H19C	0.9800
C3—H3A	0.9500	C20—H20A	0.9800
C4—C5	1.3970 (14)	C20—H20B	0.9800
C4—H4A	0.9500	C20—H20C	0.9800
C5—C6	1.4885 (14)	C21—H21A	0.9800
C6—C7	1.3866 (14)	C21—H21B	0.9800
C7—C8	1.4013 (14)	C21—H21C	0.9800
C7—H7A	0.9500		
			
C10—O1—C17	116.48 (8)	O2—C12—C11	117.43 (9)
C12—O2—C19	117.84 (8)	C13—C12—C11	120.54 (9)
C14—O3—C20	116.99 (8)	C14—C13—C12	120.38 (9)
C15—O4—C21	116.88 (8)	C14—C13—H13A	119.8
C1—N1—C5	117.40 (9)	C12—C13—H13A	119.8
C10—N2—C6	117.15 (9)	O3—C14—C13	124.30 (9)
N1—C1—C2	123.76 (10)	O3—C14—C15	115.72 (9)
N1—C1—H1A	118.1	C13—C14—C15	119.98 (9)
C2—C1—H1A	118.1	O4—C15—C16	125.54 (9)
C3—C2—C1	118.37 (10)	O4—C15—C14	115.26 (9)
C3—C2—H2A	120.8	C16—C15—C14	119.20 (9)
C1—C2—H2A	120.8	C15—C16—C11	121.96 (9)
C2—C3—C4	119.00 (10)	C15—C16—H16A	119.0
C2—C3—H3A	120.5	C11—C16—H16A	119.0
C4—C3—H3A	120.5	O1—C17—H17A	109.5
C3—C4—C5	118.62 (10)	O1—C17—H17B	109.5
C3—C4—H4A	120.7	H17A—C17—H17B	109.5
C5—C4—H4A	120.7	O1—C17—H17C	109.5
N1—C5—C4	122.85 (9)	H17A—C17—H17C	109.5
N1—C5—C6	116.40 (9)	H17B—C17—H17C	109.5
C4—C5—C6	120.73 (9)	N3—C18—C9	178.32 (12)
N2—C6—C7	123.03 (9)	O2—C19—H19A	109.5
N2—C6—C5	116.20 (9)	O2—C19—H19B	109.5
C7—C6—C5	120.77 (9)	H19A—C19—H19B	109.5
C6—C7—C8	120.21 (9)	O2—C19—H19C	109.5
C6—C7—H7A	119.9	H19A—C19—H19C	109.5
C8—C7—H7A	119.9	H19B—C19—H19C	109.5
C7—C8—C9	116.76 (9)	O3—C20—H20A	109.5
C7—C8—C11	121.44 (9)	O3—C20—H20B	109.5
C9—C8—C11	121.80 (9)	H20A—C20—H20B	109.5
C8—C9—C10	118.53 (9)	O3—C20—H20C	109.5
C8—C9—C18	123.23 (9)	H20A—C20—H20C	109.5
C10—C9—C18	118.06 (9)	H20B—C20—H20C	109.5
N2—C10—O1	120.08 (9)	O4—C21—H21A	109.5
N2—C10—C9	124.32 (9)	O4—C21—H21B	109.5
O1—C10—C9	115.60 (9)	H21A—C21—H21B	109.5
C12—C11—C16	117.94 (9)	O4—C21—H21C	109.5
C12—C11—C8	122.14 (9)	H21A—C21—H21C	109.5
C16—C11—C8	119.92 (9)	H21B—C21—H21C	109.5
O2—C12—C13	121.99 (9)		
			
C5—N1—C1—C2	0.52 (16)	C8—C9—C10—O1	179.95 (9)
N1—C1—C2—C3	−0.76 (17)	C18—C9—C10—O1	4.76 (14)
C1—C2—C3—C4	0.29 (16)	C7—C8—C11—C12	−42.34 (15)
C2—C3—C4—C5	0.33 (16)	C9—C8—C11—C12	137.61 (11)
C1—N1—C5—C4	0.16 (15)	C7—C8—C11—C16	138.34 (10)
C1—N1—C5—C6	178.65 (9)	C9—C8—C11—C16	−41.70 (14)
C3—C4—C5—N1	−0.58 (16)	C19—O2—C12—C13	−11.45 (15)
C3—C4—C5—C6	−179.01 (10)	C19—O2—C12—C11	171.00 (9)
C10—N2—C6—C7	−0.63 (15)	C16—C11—C12—O2	178.04 (9)
C10—N2—C6—C5	178.74 (9)	C8—C11—C12—O2	−1.29 (15)
N1—C5—C6—N2	−179.26 (9)	C16—C11—C12—C13	0.46 (15)
C4—C5—C6—N2	−0.74 (14)	C8—C11—C12—C13	−178.87 (9)
N1—C5—C6—C7	0.12 (14)	O2—C12—C13—C14	−176.97 (10)
C4—C5—C6—C7	178.65 (10)	C11—C12—C13—C14	0.50 (16)
N2—C6—C7—C8	1.31 (16)	C20—O3—C14—C13	−0.80 (15)
C5—C6—C7—C8	−178.03 (9)	C20—O3—C14—C15	179.26 (9)
C6—C7—C8—C9	−0.94 (15)	C12—C13—C14—O3	179.36 (10)
C6—C7—C8—C11	179.02 (9)	C12—C13—C14—C15	−0.70 (16)
C7—C8—C9—C10	0.02 (14)	C21—O4—C15—C16	8.11 (16)
C11—C8—C9—C10	−179.94 (9)	C21—O4—C15—C14	−171.38 (10)
C7—C8—C9—C18	174.95 (9)	O3—C14—C15—O4	−0.60 (14)
C11—C8—C9—C18	−5.01 (16)	C13—C14—C15—O4	179.45 (9)
C6—N2—C10—O1	−179.62 (9)	O3—C14—C15—C16	179.88 (9)
C6—N2—C10—C9	−0.37 (15)	C13—C14—C15—C16	−0.07 (15)
C17—O1—C10—N2	6.18 (14)	O4—C15—C16—C11	−178.40 (10)
C17—O1—C10—C9	−173.13 (9)	C14—C15—C16—C11	1.06 (16)
C8—C9—C10—N2	0.67 (16)	C12—C11—C16—C15	−1.25 (15)
C18—C9—C10—N2	−174.53 (10)	C8—C11—C16—C15	178.09 (10)

### Hydrogen-bond geometry (Å, º)

*Cg*_3_ is the centroid of the C11–C16 ring.

**Table:**

*D*—H···*A*	*D*—H	H···*A*	*D*···*A*	*D*—H···*A*
C20—H20*C*···N3^i^	0.98	2.59	3.3774 (17)	138
C1—H1*A*···*Cg*3^ii^	0.95	2.89	3.7062 (13)	145

Symmetry codes: (i) −*x*+1, *y*+1/2, −*z*+1/2; (ii) −*x*, *y*+1/2, −*z*+1/2.

## References

[bb1] Al-Jaber, N. A., Bougasim, A. S. A. & Karah, M. M. S. (2012). *J. Saudi Chem. Soc* **16**, 45–53.

[bb2] Allen, F. H., Kennard, O., Watson, D. G., Brammer, L., Orpen, A. G. & Taylor, R. (1987). *J. Chem. Soc. Perkin Trans. 2*, pp. S1–19.

[bb3] Brandt, W., Mologni, L., Preu, L., Lemcke, T., Gambacorti-Passerini, C. & Kunick, C. (2010). *Eur. J. Med. Chem.* **45**, 2919–2927. 10.1016/j.ejmech.2010.03.017 20409618

[bb4] Bruker (2005). *APEX2*, *SAINT* and *SADABS* Bruker AXS Inc., Madison, Wisconsin, USA.

[bb5] Chantrapromma, S., Fun, H.-K., Suwunwong, T., Padaki, M. & Isloor, A. M. (2010). *Acta Cryst.* E**66**, o79–o80. 10.1107/S1600536809051861 PMC2980143 21580178

[bb6] El-Sayed, H. A., Moustafa, A. H., Haikal, A. E.-F. Z., Abu-El-Halawa, R. & Ashry, E. S. H. E. (2011). *Eur. J. Med. Chem.* **46**, 2948–2954. 10.1016/j.ejmech.2011.04.019 21531049

[bb7] Ji, J., Bunnelle, W. H., Anderson, D. J., Faltynek, C., Dyhring, T., Ahring, P. K., Rueter, L. E., Curzon, P., Buckley, M. J., Marsh, K. C., Kempf-Grote, A. & Meyer, M. D. (2007). *Biochem. Pharmacol.* **74**, 1253–1262. 10.1016/j.bcp.2007.08.010 17854775

[bb8] Kim, K.-R., Rhee, S.-D., Kim, H. Y., Jung, W. H., Yang, S.-D., Kim, S. S., Ahn, J. H. & Cheon, H. G. (2005). *Eur. J. Pharmacol.* **518**, 63–70. 10.1016/j.ejphar.2005.05.030 16106524

[bb9] Koner, R. R., Sinha, S., Kumar, S., Nandi, C. K. & Ghosh, S. (2012). *Tetrahedron Lett.* **53**, 2302–2307.

[bb10] Macrae, C. F., Edgington, P. R., McCabe, P., Pidcock, E., Shields, G. P., Taylor, R., Towler, M. & van de Streek, J. (2006). *J. Appl. Cryst* **39**, 453–457.

[bb11] Sheldrick, G. M. (2008). *Acta Cryst.* A**64**, 112–122. 10.1107/S0108767307043930 18156677

[bb12] Spek, A. L. (2009). *Acta Cryst.* D**65**, 148–155. 10.1107/S090744490804362X PMC2631630 19171970

[bb13] Suwunwong, T., Chantrapromma, S. & Fun, H.-K. (2011). *Chem. Pap.* **65**, 890–897.

[bb14] Suwunwong, T., Chantrapromma, S. & Fun, H.-K. (2012). *Acta Cryst.* E**68**, o2812–o2813. 10.1107/S1600536812036276 PMC3435837 22969683

[bb15] Westrip, S. P. (2010). *J. Appl. Cryst.* **43**, 920–925.

[bb16] Zhou, W.-J., Ji, S.-J. & Shen, Z.-L. (2006). *J. Organomet. Chem.* **691**, 1356–1360.

